# Enduring disruption of reward and stress circuit activities by early-life adversity in male rats

**DOI:** 10.1038/s41398-022-01988-w

**Published:** 2022-06-16

**Authors:** Sophia C. Levis, Matthew T. Birnie, Jessica L. Bolton, Christina R. Perrone, Johanna S. Montesinos, Tallie Z. Baram, Stephen V. Mahler

**Affiliations:** 1 Department of Anatomy & Neurobiology, University of California Irvine, Irvine, CA USA; 2 Department of Neurobiology & Behavior, University of California Irvine, Irvine, CA USA; 3 Department of Pediatrics, University of California Irvine, Irvine, CA USA; 4 Neuroscience Institute, Georgia State University, Atlanta, GA USA

**Keywords:** Molecular neuroscience, Addiction, Psychiatric disorders

## Abstract

In humans, early-life adversity (ELA) such as trauma, poverty, and chaotic environment is linked to increased risk of later-life emotional disorders including depression and substance abuse. These disorders involve underlying disruption of reward circuits and likely vary by sex. Accordingly, we previously found that ELA leads to anhedonia for natural rewards and cocaine in male rodents, whereas in females ELA instead increases vulnerability to addiction-like use of opioid drugs and palatable food. While these findings suggest that ELA-induced disruption of reward circuitry may differ between the sexes, the specific circuit nodes that are influenced by ELA in either sex remain poorly understood. Here, in adult male Sprague-Dawley rats, we ask how ELA impacts opioid addiction-relevant behaviors that we previously tested after ELA in females. We probe potential circuit mechanisms in males by assessing opioid-associated neuronal activation in stress and reward circuit nodes including nucleus accumbens (NAc), amygdala, medial prefrontal cortex (mPFC), and paraventricular thalamus. We find that ELA *diminishes* opioid-seeking behaviors in males, and alters heroin-induced activation of NAc, PFC, and amygdala, suggesting a potential circuit-based mechanism. These studies demonstrate that ELA leads to behavioral and neurobiological disruptions consistent with anhedonia in male rodents, unlike the increased opioid seeking we previously saw in females. Our findings, taken together with our prior work, suggest that men and women could face qualitatively different mental health consequences of ELA, which may be essential for individually tailoring future intervention strategies.

## Introduction

Early-life adversity (ELA) related to poverty, trauma, and a chaotic environment affects over 30% of children in the United States [1]. In humans, ELA is associated with the development of emotional disorders such as depression and substance use later in life, suggestive of reward circuit dysfunction [2–16]. Anhedonia, or lack of pleasure from or interest in typically enjoyable activities, is a common feature of many of these disorders [14, 17–20]. Accordingly, using the limited bedding and nesting (LBN) model of ELA in male rats, we previously found that ELA causes anhedonia for natural rewards such as palatable food and social play, and even for the drug cocaine [21–25]. In contrast to males, the consequences of ELA in females were quite different: ELA-reared adult female rats showed enhanced vulnerability to addiction-like reward-seeking, including enhanced motivation to pursue addictive opioid drugs as well as palatable foods [26]. These outcomes may reflect ELA-induced disruption of stress and reward circuit development, yet the biological basis of these functional disruptions remains unclear.

Thus, in both males and females, ELA leads to behavioral changes suggesting an altered function of brain stress and reward circuits [14, 15, 21, 22, 26–28]. However, the specific circuit nodes that are influenced by ELA, and how resulting changes in brain structure and function might underlie behavioral responses to opioid drugs [29–34] remain unclear. Here, we focus on effects of ELA on opioid addiction-relevant behaviors in males, following up on our prior work in females [26]. We first examined several complementary assays of opioid-seeking behaviors, including opioid self-administration, relapse-like behavior, and economic demand elasticity. To begin to address the underlying mechanisms, we quantified heroin-induced c-Fos expression in reward and stress-related brain regions that have been implicated in anhedonia, drug-seeking, and stress, namely nucleus accumbens (NAc), amygdala, prefrontal cortex (PFC), and paraventricular thalamus (PVT) [35–39]. Because NAc is thought to integrate information from reward and stress-related afferent inputs, we also tested the effects of ELA on heroin-induced activation in NAc-projecting cells in these regions.

In contrast to our prior discovery of pro-opioid addiction effects of ELA in females, our findings here demonstrate that in males, ELA blunts certain aspects of opioid reward in a manner suggesting anhedonia for otherwise pro-hedonic opioid drugs. Further, we find that these blunted behaviors may involve altered opioid-induced neuronal activation of specific nodes within reward and stress circuits.

## Materials and methods

### Animals

Primiparous, timed-pregnant Sprague-Dawley rats were obtained from Envigo (Livermore, CA) on gestation day 15, and maintained in an uncrowded, quiet animal facility room on a 12-h light/dark cycle. Parturition was checked daily, and the day of birth was considered postnatal day (PD) 0. On PD2, pups and dams were assigned to ELA or control (CTL) groups (10–12 pups per dam, sex balanced) and housed under these conditions through PD9, as described below. Rats were weaned at PD21 and housed by sex in groups of 2-3, under a 12-h reverse light cycle. Food and water were available ad libitum throughout all experiments. ELA (*n* = 17) and CTL (*n* = 13) males from six total dams and in roughly equal number from each litter (*n* = 4–6/litter) were used for these experiments. Sample sizes were chosen based on observed effect sizes in our prior reports [22, 26]. No clear differences due to the number of cagemates were seen on behavioral outcomes, though these studies were not designed to test this variable. These ELA and behavioral testing procedures are the same as those from our prior report in females [26]. Randomization of test order of behavioral tasks was not used in the design of these studies, and experimenters were not blind to experimental group during behavioral testing. All procedures were approved by the University of California Irvine Institutional Animal Care and Use Committee and conducted in accordance with the National Institutes of Health guide for the care and use of laboratory animals.

### The limited bedding and nesting (LBN) model of early-life adversity

On PD2, pups from at least two litters at a time were gathered and assigned at random to each dam in equal numbers of male and female (10–12 pups per dam) to prevent the potential confounding effects of genetic variables, sex ratio, or litter size. Dams and pups were transferred to LBN or CTL cages, as described previously [21, 22, 40]. LBN rats were transferred to cages fitted with a plastic-coated aluminum mesh platform sitting ∼2.5 cm above the cage floor. Bedding only sparsely covered the cage floor under the platform, and one-half of a 24.2 cm 23.5 cm paper towel was provided for nesting material. CTL dams and pups were placed in cages containing a standard amount of bedding (∼0.33 cubic feet of corn cob) without a platform, and one full paper towel. Cages remained undisturbed during PD2-9. Throughout this epoch, maternal behaviors, which may constitute a source of stress in infant rats, were monitored as previously described [24, 40, 41]. On PD10, animals were all transferred to CTL condition cages, causing maternal behaviors to normalize rapidly, and for any stress to dissipate in the pups [41].

### Intravenous catheter surgery

At approximately PD70, rats were anesthetized with isoflurane (2–2.5%) and chronic indwelling catheters were inserted into the right jugular vein. Meloxicam (1 mg/kg, i.p.) for postsurgical analgesia, and prophylactic antibiotic cefazolin (0.2 ml, i.v.; 10 mg/0.1 ml) were given intraoperatively. After 5 days of recovery, catheters were flushed daily following each opioid self-administration session with cefazolin (10 mg/0.1 ml) and a heparin lock solution (10 U/0.1 ml) to maintain catheter patency.

### Drugs

Heroin (diacetylmorphine) HCl was provided by the National Institute on Drug Abuse (NIDA) Drug Supply Program (Research Triangle Park, NC, USA) or Cayman Chemical Company (Ann Arbor, MI, USA), and remifentanil HCl was provided by the NIDA Drug Supply Program. All drugs were dissolved in sterile 0.9% saline for experimental use.

### Behavioral/functional tests

Heroin self-administration, extinction, reinstatement, and remifentanil demand tests were all performed in the same rats (*n* = 7 ELA, 8 CTL). Two rats were excluded from remifentanil demand analyses: one (CTL) as a statistical outlier for the demand elasticity task (α) (Grubbs outlier test Alpha = 0.05) and one (ELA) due to catheter failure leaving six ELA and seven CTL rats in remifentanil demand analyses.

#### Heroin self-administration

Self-administration training and testing took place in Med Associates operant chambers in sound-attenuating boxes, as described previously [26]. Rats received daily 2-h self-administration sessions, when pressing on the “active” lever (AL) yielded a heroin infusion of 0.1 mg/kg (acquisition; days 1–3) or 0.05 mg/kg (training; days 4–17). Heroin infusions were accompanied by concurrent 2.9-kHz tone and lever light illumination for 3.6 s. A 20 s timeout period (signaled by turning off the house light) followed each infusion/cue presentation, during which additional lever presses were recorded but had no consequence. Pressing on the second “inactive” lever (IL) was recorded but had no consequence.

#### Heroin extinction and reinstatement

Following heroin self-administration, rats received extinction training for a minimum of 7 days, or until extinction criterion (2 consecutive days <20 AL presses) was met. Lever presses were recorded but had no consequence. Upon meeting the extinction criterion, rats underwent a 2-h cue-induced reinstatement test, during which one presentation of the drug-paired cues was delivered 10 s after the start of the session, then AL presses yielded additional cue presentations. Rats then underwent a minimum of 2 extinction training days until the extinction criterion was re-attained, after which they underwent drug/vehicle-primed reinstatement tests. Rats received an experimenter-administered injection of heroin (0.25 mg/kg, s.c [26, 42]) or saline immediately before the start of the 2-h session. Lever presses were recorded but did not yield additional heroin or cues. All animals received both heroin and saline on separate days in counterbalanced order. Again, 2+ additional extinction training days occurred between primed reinstatement tests to re-establish the extinction criterion. Following reinstatement testing, catheter patency was confirmed using intravenous methohexital (0.1–0.2 ml, 5 mg/ml). Rats with catheter failure (*n* = 1) were re-catheterized in the left jugular vein and allowed to recover for five days before starting the behavioral economic procedures.

#### Behavioral economic thresholding procedure

**Fig. 1:**
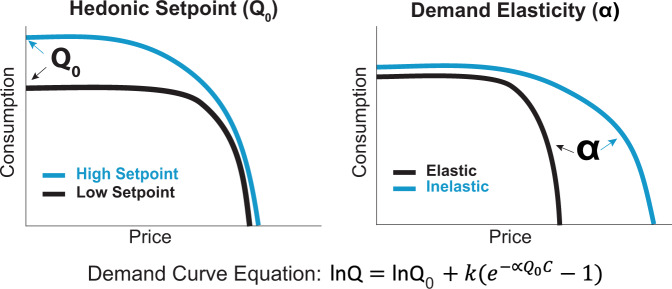
Demand characteristics in sample curves. Schematic representation of sample demand curves generated from hypothetical response patterns reflecting differences in hedonic setpoint (*Q*_*0*_) and demand elasticity (α). Drug intake was determined at each response requirement and consumption data were modeled with an exponential demand equation: ${{{\mathrm{lnQ}}}} = {{{\mathrm{ln}}}}Q_0 + k(e^{ - \propto Q_0C} - 1)$ [44, 60], where Q = consumption, C = unit cost, k is a scalar constant for consumption range, α = demand elasticity, and Q_0_ = extrapolated intake at zero effort. This process yields a demand curve fitted to consumption at each price, from which variables corresponding to the hedonic setpoint (Q_0_) and motivation (α) are derived.

Rats were trained on a previously described within-session economic thresholding procedure [22, 26, 43–46]. This behavioral tool, variations of which can be implemented in both rodents and humans [44, 47–50], allows for simultaneously measuring both consummatory and motivational aspects of reward by systematically increasing the cost of a particular commodity or substance, such as a drug or food reinforcer. By mathematically generating a demand curve based on lever press responses at increasing costs (as described below and illustrated in Fig. 1), we determine the hedonic setpoint (reflected by preferred consumption at low effort) and motivation, otherwise referred to as demand elasticity (how much effort is expended to obtain the drug when the cost is high).

To avoid undesired effects of drug satiety on responding due to the long half-life of heroin, this task was adapted for use with the short-acting fentanyl derivative remifentanil [26, 43], chosen for its rapid clearance from blood and tissues that allows for precise dose titration [51]. Each AL press delivered remifentanil and a concurrent cue presentation. The duration of each cue/infusion (and hence the amount of drug per infusion) was decreased in successive 10-min bins across the 110-min session, signaling that rats were required to exert increasing effort (i.e., pay a higher price) to obtain a desired amount of drug. The resulting doses during each bin are as follows: 2, 1, 0.6, 0.3, 0.2, 0.1, 0.06, 0.03, 0.02, 0.01, and 0.006 µg/infusion [26, 43]. Drug intake was determined at each response requirement and consumption data was modeled with an exponential demand equation${{{\mathrm{lnQ}}}} = {{{\mathrm{ln}}}}Q_0 + k(e^{ - \propto Q_0C} - 1)$ [22, 26, 43, 44], where Q = consumption, C = unit cost, k is a scalar constant for the consumption range, α = demand elasticity, and Q_0_ = extrapolated intake at zero effort. This process yields a demand curve fitted to consumption at each bin, from which variables correspond to the hedonic setpoint (Q_0_, reflecting the hedonic value of remifentanil extrapolated to price 0) and motivation (α, reflecting demand elasticity or sensitivity to increasing price) are derived. Rats were trained on the threshold procedure for 14 days, and behavior derived from the average of the last 5 days was used in final analyses. As in prior reports using this approach [22, 43, 44, 46, 52], the highest-effort bins, in which behavior was sporadic or absent, were removed to better fit demand curves.

#### NAc projection tracing

Following the behavioral testing described above, rats received stereotaxic unilateral NAc pressure injections of ~40 nL of cholera toxin beta subunit (CTb, 0.5%) aimed at the border of NAc core and shell (mm from bregma: AP + 1.35; ML + or −1.55; DV −7.6), using a Picospritzer (General Valve, Inc.) via a glass pipette (internal diameter ~12 mm). Pipettes were left in place for 10 min following the infusion to minimize back-diffusion up the pipette track. Rats were anesthetized and perioperative medications administered as described above. They were allowed to recover for a minimum of 5–7 days prior to acute heroin challenge and the resulting c-Fos expression experiments, a duration that does not appear to affect c-Fos expression, or cause signs of cytotoxicity in prior comparable work [53, 54].

#### Heroin-induced neuronal activation

Rats were injected with heroin (0.25 mg/kg, s.c.) and immediately placed in a novel environment (43 cm × 43 cm × 30.5 cm chamber with transparent walls and without bedding or food/water) for 1 h, then returned to home cages. This non-sedating dose and route of administration was chosen due to its reinforcing and reinstating properties [26, 42]. 120 min following the heroin injection, animals were sacrificed for c-Fos analysis, as described in the below sections.

To pinpoint if ELA-induced changes in heroin-induced neuronal activation depended upon a prior history of opioid exposure, a separate group of handled, age-matched, but otherwise experimentally naïve rats (*n* = 10 ELA, 5 CTL) underwent the same CTb surgeries, a single acute heroin challenge, and sacrifice.

### c-Fos & CTb studies

#### Tissue preparation

Rats were transcardially perfused with chilled 0.9% saline followed by 4% paraformaldehyde under deep anesthesia. Brains were removed and postfixed overnight, then cryoprotected in a 20% sucrose-azide solution. Frozen 40-μm coronal sections were collected on a cryostat and stored in phosphate-buffered saline (PBS) with sodium azide at 4 °C for subsequent immunohistochemistry analyses.

#### Immunohistochemistry

An avidin-biotin complex (ABC)-amplified, diaminobenzidine (DAB) reaction was conducted to visualize heroin-induced c-Fos and CTb expression. Endogenous peroxidase was blocked with 0.3% H_2_O_2_, then 3% normal donkey serum (Vector Laboratories, Burlingame, CA) in PBS containing 0.3% Triton-X. Sections were incubated for 16 h at room temperature polyclonal rabbit anti-c-Fos, washed, then incubated for 2 h in biotinylated donkey anti-rabbit IgG (1:500, Jackson Laboratories, West Grove, PA). After ABC amplification (90 min), Fos was visualized in blue/black with DAB in Tris buffer with 0.01% H_2_ O_2_ and Nickel Ammonium Chloride (0.04%; Vector Laboratories). Sections from brains with appropriate CTb placement were further processed to visualize somatic CTb in brown (without nickel intensification). Tissue was incubated at room temperature in goat anti-CTb (1:10,000, Millipore) for 16 h, followed by biotinylated donkey anti-goat (1:500; Jackson Laboratories), ABC amplification, and DAB reaction. Sections were mounted and coverslipped in Permount medium.

CTb injection sites were visualized using fluorescent immunohistochemistry. Sections were incubated in goat anti-CTb (1:5000; Millipore) primary antibody overnight at room temperature. After washing, sections were incubated in donkey anti-goat Alexa Fluor 488 (1:500; Invitrogen) at room temperature for 4 h, washed, then mounted and coverslipped with Fluoromount mounting medium and stored at 4 °C until photographed. CTb injection sites were quantified at 5X magnification and compared with a rat brain atlas (Paxinos and Watson, 2007) to localize tracer injections (Fig. 4A). Most injections spread to both shell and core of NAc. Brains with misplaced CTb injections or leakage beyond NAc borders were not used for CTb quantification, and were only used for quantifying heroin-induced c-Fos expression.

Initial rounds of Fos IHC were conducted using Millipore polyclonal rabbit anti-c-Fos (#ABE457; 1:5,000), however due to stock shortages, later rounds of IHC were conducted using Abcam polyclonal rabbit anti-c-Fos (#GR3293718-1, 1:10,000). The two antibodies were compared head-to-head in neighboring NAc sections from the same brains (*n* = 8 brains; two sections per brain per antibody), and both qualitative appearance and the cell counts did not differ significantly between the two antibodies (Fig. S1; total NAc cell density Millipore vs. Abcam *t*_30_ = 0.3442, *P* = 0.7331*;* Pearson correlation between antibodies tested on tissue from the same rats: *r* = 0.6637, *P* = 0.0051), so data from both antibodies were combined.

Of the opioid-experienced rats, one CTL brain was excluded due to incomplete exsanguination during perfusion for a total of seven ELA and seven CTL rats for Fos analysis. An additional two CTL and one ELA rats were excluded from CTb analyses due to CTb infusion localized outside NAc. Of the opioid-naïve rats, one CTL and three ELA rats were excluded from CTb analyses due to misplaced infusion outside NAc. One additional CTL rat was excluded from only PFC CTb analyses due to tissue damage during processing.

#### Imaging and Fos analysis

Images of structures quantified for Fos/CTb were taken at 10X magnification on a Leica DM4000B microscope with stage automation, and stitched using Stereo Investigator (SI) software (MicroBrightfield). Three to four coronal sections per structure from comparable regions in each animal were quantified bilaterally by a trained, blinded observer. Brain region borders were delineated based on a brain atlas (Paxinos and Watson, 2007). The coordinate range sampled from each structure is as follows (mm relative to bregma): PFC: +3.24 - +3.00; NAc: +2.28–+1.44; CeA; −2.28 to −2.76; BLA: −2.28 to −2.92; PVT: −2.16 to −3.00. NAc and PFC were further delineated into sub-structures (NAc core and shell and infralimbic and prelimbic PFC). Fos+ neurons were identified using the SI particle counter tool, and Fos density (Fos/mm^2^) was computed for each sample as in our prior reports [22]. On sections also stained for CTb, the SI particle counter tool was also used to count Fos on the hemisphere contralateral to the injection site where few CTb+ cells were present, and Fos counts were manually checked by a trained observer to avoid misidentification of cells. CTb+ only, Fos+ only, and dual-labeled (CTb+ and Fos+) neurons were quantified manually using ImageJ from the hemisphere ipsilateral to the CTb injection. To normalize variability in precise CTb injection volume and localization across animals, the percentage of NAc-projecting (CTb+) cells that were also Fos+ was used for primary analyses [55, 56].

#### Analysis approach

Statistical analyses were performed using GraphPad Prism software. Independent samples *t*-tests were used to determine the effects of ELA on heroin consumption, extinction persistence (days until extinction criterion), and demand characteristics for remifentanil (Q_0_, α). Extinction persistence was further analyzed for “survival” of seeking behavior (i.e., percentage of rats in each group failing to meet extinction criterion on each training day), and Kaplan–Meier plots were compared using the log-rank test [57]. For analyses of ELA effects on within-subjects variables (e.g., saline/heroin-primed reinstatement), two-way repeated-measures ANOVAs were used. Between-subjects two-way ANOVAs were used to test interactions of ELA with opioid experience on Fos. Following significant ANOVA main effects and/or interactions, Bonferroni post hoc tests were used to characterize the nature of effects. Independent samples *t*-tests were also used to describe ELA effects on heroin-induced neuronal activation in combined groups of naïve and experienced animals. Effect sizes for significant and trending effects are reported as eta squared (η^2^) for *t*-tests and partial eta squared (η_p_^2^) for ANOVA. Compared groups did not statistically differ from one another in variance, accommodating assumptions of the parametric tests employed.

## Results

### Effects of ELA on drug-seeking and drug-taking behaviors

#### Heroin self-administration

**Fig. 2:**
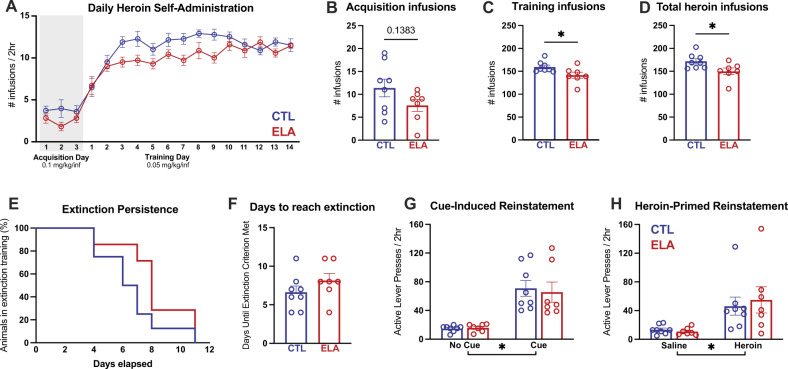
ELA reduces heroin self-administration but not does not alter extinction or relapse. **A** Average daily heroin consumption. **B** There was a trend towards reduced consumption of heroin during the high-dose acquisition phase among ELA-reared animals. ELA led to reduced consumption during the training phase (**C**) and overall consumption (**D**). Despite reduced intake, ELA did not alter extinction (**E**, **F**) or reinstatement to heroin seeking (**G**, **H**). * ELA vs CTL *P* < 0.05. *n* = 8 CTL/7 ELA.

As shown in Fig. 2, adult male rats with a history of ELA (ELA rats) self-administered fewer total heroin infusions than their control counterparts over the course of heroin self-administration (SA) (Fig. 2D; *t*_13_ = 2.386, *P* = 0.0329, η^2^ = 0.3046). During the initial 3 days of heroin SA (acquisition; 0.1 mg/kg/infusion [inf]), ELA rats non-significantly trended toward consuming less heroin than controls (Fig. 2B; *t*_13_ = 1.579, *P* = 0.1383, η^2^ = 0.1609), but after transitioning to a lower heroin dose (training; 0.05 mg/kg/inf) for the following 14 days, consumption was significantly lower in ELA rats than controls (Fig. 2C; *t*_13_ = 2.341, *P* = 0.0359, η^2^ = 0.2965), though this difference appeared to diminish during the later training days (Fig. 2A).

#### Heroin extinction and reinstatement

Heroin extinction did not distinguish ELA and CTL males. Both groups required the same amount of training to reach extinction criteria (Fig. 2F; *t*_13_ = 1.257, *P* = 0.2308), and the probability of achieving extinction criterion each day was similar (Fig. 2E; Kaplan–Meier probability of survival log-rank curve comparison Chi-square(df) = 2.052(1), *P* = 0.1520).

Cue-induced and heroin-primed reinstatement were apparent in both ELA and CTL rats (Fig. 2G, H; main effect of light and tone cue vs. no-cue: *F*_(1,13)_ = 39.53, *P* < 0.0001, η_p_^2^ = 0.7525; main effect of heroin vs. saline: *F*_(1,13)_ = 12.12, *P* = 0.0041, η_p_^2^ = 0.4825). ELA did not alter cue reinstatement (main effect of rearing condition: *F*_(1,13)_ = 0.05063, *P* = 0.8255; interaction: *F*_(1,13)_ = 0.1450, *P* = 0.7095) or heroin-primed reinstatement (main effect of rearing condition: *F*_(1,13)_ = 0.07819, *P* = 0.7842; interaction: *F*_(1,13)_ = 0.2660, *P* = 0.6147).

#### Intra-session heroin self-administration timecourse

**Fig. 3:**
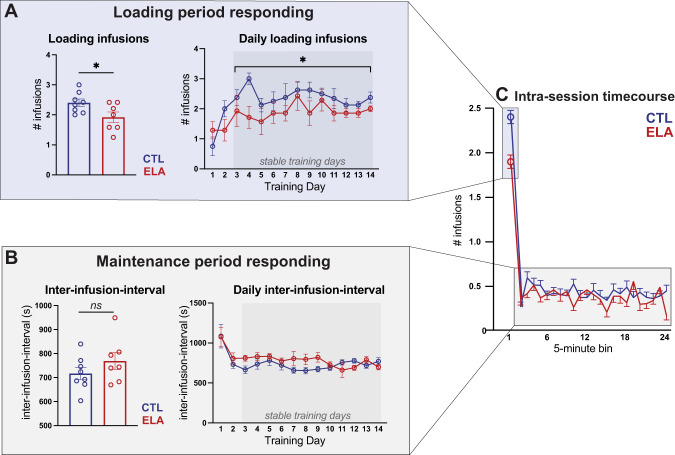
ELA specifically blunts loading phase heroin consumption. **A** ELA rats consumed fewer infusions during the first 5 min “loading period” of the 2 h session across stable training days (days 3–14). **B** Average inter-infusion-interval was not different between ELA and CTL, but there was a significant ELA x training day interaction on stable training days (days 3–14). **C** Average consumption across the 2 h session. * ELA vs CTL *P* < 0.05. *n* = 8 CTL/7 ELA.

To assess whether the above findings may be a result of differences in drug sensitivity, malaise/sedation, development of pharmacological tolerance or sensitization, or other potential non-hedonic factors, we analyzed the pattern of heroin self-administration behavior within the 2 h training sessions, following establishment of stable responding (training days 3–14). Rats showed characteristic “drug loading” in the first 5 min of each session, followed by slower “maintenance” responding the remainder of the session (Fig. 3C). Average drug consumption during the loading phase was significantly lower in ELA than control rats (Fig. 3A; *t*_13_ = 2.321, *P* = 0.0372, η^2^ = 0.2931), an effect that was constant across stable training days (main effect of rearing condition: *F*_(1,13)_ = 5.389, *P* = 0.0372, η_p_^2^ = 0.2931; main effect of day: *F*_(11,143)_ = 0.97, *P* = 0.4765; interaction: *F*_(11,143)_ = 0.5971, *P* = 0.8288). There was no effect of ELA on the average inter-infusion-interval (I-I) during the remainder of the session (Fig. 3B; main effect of rearing condition across stable training days: *F*_(1,13)_ = 1.499, *P* = 0.2426). In addition, there was a significant ELA x day interaction, whereby the I-I tended to decrease as training progressed in ELA rats but increased over training in controls (days 9–14; main effect of training day: *F*_(11,143)_ = 0.9357; *P* = 0.5082; rearing x day interaction: *F*_(11,143)_ = 2.241, *P* = 0.0086, η_p_^2^ = 0.1470;). Finally, there was no effect of ELA on inactive lever presses (main effect of rearing condition: *F*_(1,13)_ < 0.01, *P* = 0.99; main effect of training day: *F*_(16,208)_ = 0.4227, *P* = 0.9756; interaction: *F*_(16,208)_ = 1.268, *P* = 0.2204), suggesting no overt ELA-induced locomotor deficits, as expected [22, 24, 58, 59].

#### Demand for remifentanil

**Fig. 4:**
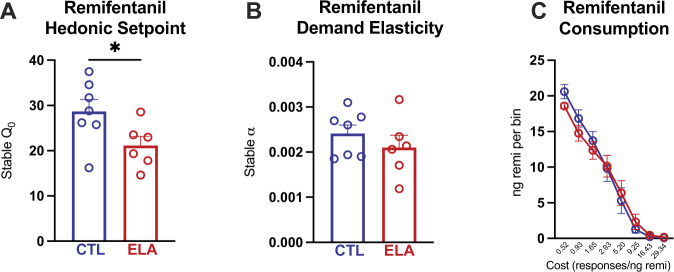
ELA reduces hedonic setpoint for opioids, but demand elasticity is preserved. **A** Hedonic setpoint or consumption at low effort is reduced by ELA. **B** Demand elasticity or motivation to obtain drug at high cost, is not changed by ELA. **C** Average consumption at each price. * ELA vs. CTL *P* < 0.05. *n* = 7 CTL/6 ELA.

Analysis of stable responses for remifentanil revealed that ELA males had a reduced “hedonic setpoint” for the drug (Q_0_). Q_0_, a value extrapolated from the animal’s calculated demand curve, is a parameter that reflects consumption at low cost or when the drug is essentially “free” [44, 60] (Fig. 4A; *t*_11_ = 2.213, *P* = 0.0490, η^2^ = 0.3080), in accord with our published finding for cocaine in male ELA rats [22]. In contrast to the hedonic setpoint, ELA did not alter demand elasticity (α) for remifentanil (Fig. 4B; *t*_11_ = 0.9560, *P* = 0.3596), indicating that ELA and control males were similarly sensitive to increasing “price” of the drug, as an increasing effort was required to maintain preferred blood levels. Notably, we previously found this indicator of motivation for remifentanil was robustly enhanced by ELA in female rats [26].

Whereas these experiments were not designed to detect sex differences, we performed a two-way ANOVA directly comparing our previously published data in females [26] with the current data in males. For hedonic setpoint (*Q*_*0*_), there was a main effect of sex (*F*_1,39_ = 24.43, *P* < 0.001, η_p_^2^ = 0.3851) and of rearing condition (*F*_1,39_ = 7.051, *P* = 0.0114, η_p_^2^ = 0.1531), and a trend toward interaction of these variables (*F*_1,39_ = 3.420; *P* = 0.0720; η_p_^2^= 0.0806). Notably, ELA reduced the hedonic setpoint only in males (ELA vs CTL: males *P* = 0.0207; females *P* = 0.9341). For demand elasticity (α), there was a main effect of rearing condition across the sexes (*F*_1,39_ = 4.328, *P* = 0.0441, η_p_^2^ = 0.0999), though this effect survived post hoc multiple comparison correction only in females (ELA vs CTL: females *P* = 0.0105; males *P* > 0.999). There was no significant effect of sex on α (*F*_1,39_ = 2.064; *P* = 0.1588) and no sex x rearing condition interaction (*F*_1,39_ = 1.356; *P* = 0.2513). These comparisons are thus consistent with, though do not prove, the testable hypothesis that ELA differentially affects opioid reward in males and females.

### Heroin-induced neuronal activation

#### Effects of ELA

**Fig. 5:**
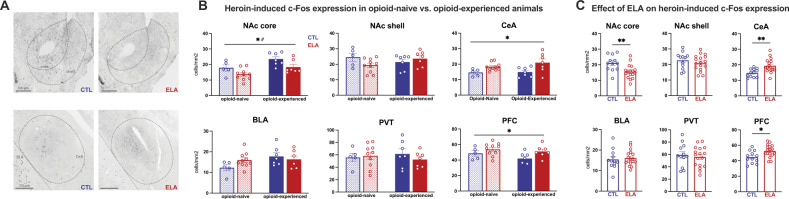
ELA alters heroin-induced neuronal activation in a manner independent of prior opioid experience. ELA leads to blunted activation of NAc core but not the shell and leads to aberrant activation in CeA and PFC. ELA did not alter heroin-induced activity in BLA or PVT. **A** Representative images of c-Fos expression in NAc and CeA. **B** Heroin-induced c-Fos expression (cell density) in all animals separated by opioid history. **C** Heroin-induced c-Fos expression in all animals collapsed across opioid exposure conditions. *ELA vs. CTL *P* < 0.05; **ELA vs. CTL *P* < 0.005; # opioid-naive vs. opioid-experienced *P* < 0.05. experienced *n* = 7 CTL/7 ELA; naïve *n* = 5 CTL/10 ELA.

ELA led to several changes in the pattern of heroin-induced Fos expression, when analyzed regardless of prior opioid experience. Specifically, ELA blunted heroin-induced c-Fos expression in NAc core (Fig. 5C; *t*_27_ = 3.108, *P* = 0.0044, η^2^ = 0.2634) but not shell (Fig. 5C; *t*_27_ = 0.8425, *P* = 0.4069). In contrast, ELA increased heroin-induced Fos expression in CeA (Fig. 5A; *t*_27_ = 3.188, *P* = 0.0036, η^2^ = 0.2734) and PFC (Fig. 5C; *t*_27_ = 2.675, *P* = 0.0125, η^2^ = 0.2095). The selectivity of these effects of ELA are apparent from the lack of changes in BLA (Fig. 5C; *t*_27_ = 0.4239, *P* = 0.6750) or PVT (Fig. 5C; *t*_27_ = 0.4901, *P* = 0.6280).

#### Influence of prior opioid exposure

Next, we examined the impact of prior chronic opioid self-administration history on heroin-induced Fos in ELA or control rats. In opioid-experienced rats, heroin-induced NAc core Fos expression was higher than in previously opioid-naïve rats (Fig. 5B; main effect of opioid experience: *F*_(1,25)_ = 11.28, *P* = 0.0025, η_p_^2^ = 0.3109). There was no experience x rearing condition interaction (*F*_(1,25)_ = 0.1024, *P* = 0.7516). The main effect of rearing condition described above persisted in this analysis: *F*_(1,25)_ = 8.868, *P* = 0.0064, η_p_^2^ = 0.2618). In NAc shell, ELA rats trended toward having lower Fos when opioid-naive, relative to when opioid-experienced Fig. 5B; interaction: *F*_(1,25)_ = 3.840, *P* = 0.0613; η_p_^2^ = 0.1331), and there was no main effect of rearing condition (*F*_(1,25)_ = 0.6295, *P* = 0.4350) or opioid exposure observed (*F*_(1,25)_ = 0.1325, *P* = 0.7190). In BLA, opioid-experienced rats showed a near-significant increase in heroin-induced Fos relative to naïve rats (5B; main effect of opioid experience: *F*_(1,25)_ = 3.893, *P* = 0.0596; η_p_^2^ = 0.1347), with no main effect of rearing group (Fig. 5B; *F*_(1,25)_ = 0.6029, *P* = 0.4448*)*. A nonsignificant trend towards an ELA × opioid experience interaction was also observed (Fig. 5B; *F*_(1,25)_ = 2.906, *P* = 0.1007; η_p_^2^ = 0.1041).

In contrast to the effects in NAc and BLA, there was a trend towards reduced c-Fos expression in PFC in both ELA and CTL rats following chronic opioid exposure (Fig. 5B; main effect of opioid experience: *F*_(1,25)_ = 2.389, *P* = 0.1348; η_p_^2^ = 0.0872), and no ELA × opioid experience interaction (*F*_(1,25)_ = 0.4404, *P* = 0.5130). However, the primary effect of ELA was maintained (Fig. 5B; main effect of rearing condition: *F*_(1,25)_ = 5.764, *P* = 0.0241, η_p_^2^ = 0.1874). We then analyzed infralimbic (ILC) and prelimbic (PLC) portions of PFC to determine whether the overall activation of PFC may be driven by one subregion or a sum of opposing effects in these two functionally distinct subregions. While the overall effects of ELA on c-Fos expression in PFC were of a consistent pattern across subregions, prior opioid experience altered heroin-induced activation significantly only in PLC, without as strong an effect in ILC (PLC: main effect of opioid experience *F*_(1,25)_ = 5.203, *P* = 0.0313, η_p_^2^ = 0.1723; main effect of rearing condition: *F*_(1,25)_ = 2.557, *P* = 0.1224; interaction: *F*_(1,25)_ = 1.394, *P* = 0.2488; ILC: main effect of opioid experience *F*_(1,25)_ = 1.039, *P* = 0.3183; main effect of rearing condition: *F*_(1,25)_ = 3.230, *P* = 0.0849; interaction: *F*_(1,25)_ = 0.7481, *P* = 0.3956*)*.

There was no effect of prior opioid experience in CeA of either rearing group, and no ELA × opioid experience interaction (Fig. 5B; main effect of opioid experience *F*_(1,25)_ = 1.179, *P* = 0.2879; interaction *F*_(1,25)_ = 0.6277, *P* = 0.4358). As with NAc and PFC, the main effect of ELA was maintained (Fig. 5B; *F*_(1,25)_ = 11.16, *P* = 0.0026, η_p_^2^ = 0.3086).

Finally, there was no effect of rearing condition or opioid experience in PVT (Fig. 5B; main effect of rearing condition: *F*_(1,25)_ = 0.2309, *P* = 0.6350; main effect of opioid experience: *F*_(1,25)_ < 0.01, *P* = 0.9792; interaction: *F*_(1,25)_ = 0.7464, i = 0.3958).

### Heroin-induced activation of NAc-projecting cells

**Fig. 6:**
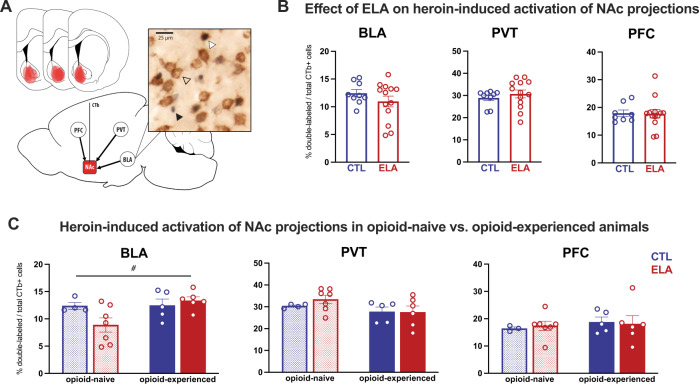
ELA does not substantially alter the neuronal response to heroin in NAc-projecting cells. **A** Diagram of NAc CTb injection placements and example of cell appearance. Black arrow = Fos+, clear arrow = CTb+; white arrow = Fos+CTb+ cell. **B** Percentage of total CTb-labeled cells that are also Fos+ in all animals collapsed across opioid exposure conditions. There was no effect of ELA on the activation of NAc-projecting cells in BLA, PVT, or PFC. **C** Percentage of total CTb-labeled cells that are also Fos+ in all animals separated by opioid history. # opioid-naive vs. opioid-experienced *P* = 0.05. experienced *n* = 5 CTL/6 ELA; naïve *n* = 4 CTL (3 CTL for PFC)/7 ELA.

The previous analyses identified ELA-induced alterations in heroin-induced Fos expression in select nodes of reward and stress circuits. Yet these nodes functionally communicate as a circuit, so we aimed to increase the resolution of our assessments by determining the effects of ELA specifically on circuit projections from these regions to NAc. Specifically, we found that ELA had no direct effect on heroin-induced activity in NAc-projecting BLA, PVT, or PFC neurons (Fig. 6B; BLA: *t*_20_ = 1.136, *P* = 0.2695; PVT: *t*_20_ = 0.7545, *P* = 0.4593; PFC: *t*_19_ = 0.09062, *P* = 0.9287). However, activation of some of these populations tended to be influenced by prior opioid exposure. For example, in NAc-projecting BLA cells, there was a main effect of opioid experience (Fig. 6C; *F*_(1,18)_ = 4.378, *P* = 0.05, η_p_^2^ = 0.1956), and a near-significant interaction with ELA (*F*_(1,18)_ = 3.961, *P* = 0.0620; η_p_^2^ = 0.1803) such that there was a lower percentage of heroin-activated NAc-projecting BLA cells after ELA in previously opioid-naïve rats, which tended to be reversed by chronic opioid exposure. There was no main effect of rearing condition on this cell population (*F*_(1,18)_ = 1.393, *P* = 0.2533). In PVT there was a trend towards reduced activity in NAc-projecting cells following opioid experience (Fig. 6C; main effect of opioid experience: *F*_(1,18)_ = 3.491, *P* = 0.0781, η_p_^2^ = 0.1624; main effect of rearing condition: *F*_(1,18)_ = 0.4052, *P* = 0.5324; interaction: *F*_(1,18)_ = 0.5323, *P* = 0.4750). In NAc-projecting PFC neurons, Fos was not influenced by prior opioid experience or ELA (Fig. 6C; main effect of rearing condition: *F*_(1,17)_ < 0.01, *P* = 0.9559; main effect of opioid experience: *F*_(1,17)_ = 0.4589, *P* = 0.5073*;* interaction: *F*_(1,17)_ = 0.1241, *P* = 0.7290). These results remained when infralimbic and prelimbic cortices were analyzed separately (*F* < 1, *P* > 0.2 for all main effects and interactions).

## Discussion

Here we show that ELA in males reduces opioid consumption behaviors, and alters heroin-induced neural activity in specific brain stress- and reward-related structures. Our principal findings are that: (1) ELA diminishes behavioral reward responses to opioids in males, apparent from blunted heroin and remifentanil taking, (2) the mechanism of the ELA-induced reduction of opioid self-administration involves altered activation of reward and stress circuit nodes including NAc, PFC, and specific amygdala nuclei, and (3) chronic exposure to opioids alters the impact of ELA on neuronal activation after acute heroin, raising intriguing speculation that this exposure might mitigate aspects of the anhedonia-like phenotype provoked by ELA in male rats.

### ELA blunts opioid self-administration and hedonic value

We found that in males, ELA produced behavioral changes consistent with anhedonia for opioids, in accordance with our prior findings of ELA-induced anhedonia for natural and cocaine rewards [21, 22] and with the recent work of Ordoñes Sanchez et al. [29], who also found that ELA imposed by LBN reduced opioid (morphine) intake in male rats. Specifically, we found that ELA males self-administered less heroin than controls, and had a lower hedonic setpoint in a within-session remifentanil economic demand task. Intriguingly, ELA appeared to reduce heroin consumption primarily during the “loading phase” of each self-administration session (first 5 min), and thereafter ELA and CTL animals titrated their intake at roughly the same rate throughout the remainder of the 2 h session. Together with reduced remifentanil hedonic setpoint (calculated based on low-effort responding in the economic demand task), these data are consistent with ELA males preferring lower levels of intake/intoxication than controls.

In contrast to ELA effects on low-effort opioid self-administration, other addiction-associated parameters (extinction, reinstatement, inelastic opioid demand) were not impacted by ELA in males, suggesting that reduced hedonic consumption does not necessarily predict suppression of other addiction-like behaviors, or preclude the possibility of developing a substance use disorder. Indeed, in humans, substance use disorders commonly co-occur with anhedonia [61]. Thus, our present findings support the notion that in males, ELA may increase risk for mental illnesses characterized by the presence of anhedonia without directly altering the risk for developing a comorbid substance use disorder.

An alternative interpretation of these findings is that, rather than causing opioid reward anhedonia, ELA may alter sensitivity to the pharmacologic properties of the drug itself. This alternative is not well-supported by our findings, as ELA did not significantly impact heroin inter-infusion-interval in the post-loading phase of daily self-administration, nor did ELA impact inactive lever pressing. Likewise, ELA and control rats responded similarly for remifentanil at higher effort requirements in the economic demand task. This likely indicates that ELA animals were not more sensitive to either the sedating or locomotor sensitizing effects of opioid drugs, but rather that they preferred to maintain lower blood levels of them. This, together with our prior work demonstrating a lack of effect of ELA on other drug-related physiological processes [22], supports the interpretation that ELA influences opioid consumption in a manner consistent with an underlying anhedonic state, manifest in their response to a variety of rewards [21, 22]. This said, an ELA effect on pharmacologic sensitivity to heroin cannot be completely ruled out, and should thus be further investigated with complementary behavioral pharmacological approaches.

### ELA may alter disparate aspects of reward-seeking in males and females

Reward-related behaviors can be either consummatory (“liking”) or motivational (“wanting”) [62] in nature, and these processes have distinct neurobiological mechanisms [44, 63–67]. The economic demand model employed here is a useful tool for simultaneously studying perturbations of these two dissociable aspects of reward [22, 26, 50, 52, 56, 68, 69]. This test capitalizes on behavioral economic theory, which stipulates that consumption of any commodity is sensitive to increasing price. Relative sensitivity to increasing price is referred to as “demand elasticity” [49]. Inelastic demand, or relative insensitivity to price, is a feature of the excessive reward-seeking associated with substance use disorders [47]. This behavior is distinct from drug intake when the required effort to attain preferred blood drug levels is very low. Specifically, consumption that persists at a high cost is more reliant on motivational processes (i.e., drug “wanting”), while low-cost drug consumption corresponds instead to hedonic value (or drug “liking”), governed by a so-called “hedonic setpoint” [47, 48, 60]. Anhedonia may therefore manifest as a decreased hedonic setpoint for a given reinforcer, independent of changes to demand elasticity. Thus, our finding of reduced remifentanil consumption only at low effort, and decreased intake of heroin only in the initial loading phase of self-administration, suggests that ELA may alter brain function in males in a manner that causes deficits specifically in hedonic aspects of opioid consumption.

The outcomes discussed in males above are distinct from our prior findings in females using the same behavioral tasks [26]. In contrast to males, ELA females had enhanced motivation to obtain opioids at high effort (inelastic demand), increased extinction responding and reinstatement, and no change in hedonic setpoint compared to controls, implicating perturbation of circuits governing motivational aspects of reward. Whereas the present studies were not designed to directly demonstrate sex differences, our observations in each individual sex, together with the trending interactions between the effects of sex and ELA on economic demand characteristics (α and *Q*_*0*_) even in our underpowered comparison, support the notion that ELA might lead to different addiction-relevant outcomes in males and females, in accord with epidemiological observations in humans [7, 10, 14, 70–72]. The basis for these differences likely involves both intrinsic functional differences in the organization and function of the reward circuit across sexes [73], as well as the potential that ELA affects brain development differentially in males and females [74, 75]. In both cases, future studies aimed at understanding how ELA alters distinct types of reward-related behaviors across sexes would provide clues into the developmental effects of ELA on reward circuitry and subsequent risk for psychiatric illness, and how these may differ by sex.

### ELA alters the balance of stress and rewards circuit activation by heroin

The nucleus accumbens is a central node of the circuit that governs reward-seeking behaviors and can be segmented into anatomically and functionally distinct regions including the shell and core [76–79]. We found that ELA leads to blunted activation of the nucleus accumbens core, but not shell in response to acute heroin, suggesting that ELA-experienced males may be less sensitive to the rewarding or reinforcing effects of opioids. This finding is again consistent with Ordoñes Sanchez et al. [29], who also observed reduced excitatory activity in NAc core of LBN-reared males. Although the NAc shell (rather than core) has been classically associated with opioid-dependent hedonic processing [80, 81], others have observed reduced c-Fos expression in the NAc core of anhedonic rats after reward consumption [82]. Therefore, inadequate activation of the NAc core in response to pleasurable stimuli like heroin may be a feature of anhedonia induced by ELA [83]. Notably, c-Fos expression in NAc following social play and cocaine is not altered by ELA [21, 22], suggesting that ELA alters brain responses to different rewards in a distinct manner [15].

In addition to blunted NAc activation, we observed aberrant activation of CeA and PFC in ELA males compared to controls. Interestingly, whereas activation of other brain structures is altered by ELA in a reward-specific manner, CeA is the only structure we observed with elevated activation in response to opioids as well as social play and cocaine [21, 22], three distinct types of rewards. This suggests that dysfunction of CeA, an important node involved in encoding and processing stress [84] as well as reward [85, 86], may represent an important mechanism by which ELA causes global reward-related deficits in males. Among the studied amygdala nuclei, these findings were specific to CeA: they were not identified in BLA. Rather, in BLA, ELA and chronic opioid experience tended to interact to affect later response to heroin, as discussed below. In PFC, ELA enhanced heroin-induced Fos overall, without affecting activity in NAc-projecting cells. Although projections from PFC into NAc mediate reward-seeking behaviors, including for opioids [87–89], this finding suggests that the PFC→NAc pathway is not overtly altered by ELA. ELA may thus instead alter other PFC neurons such as those targeting other brain regions—a possibility that should be investigated in future studies.

We did not observe any effects of ELA on heroin-associated PVT activation, nor on activation of its NAc-projecting cells, suggesting that ELA does not alter PVT control over opioid reward, despite its role in a variety of reward-related behaviors [39], including drug-seeking [35].

We note that these changes in heroin-induced c-Fos expression were observed following exposure to a novel environment, which likely has its own effects on neuronal activation, particularly in stress-related regions. While we cannot dissociate the effects of the novel environment from the effects of heroin in this study, we have previously observed no difference in anxiety-like behaviors in male rats following ELA using elevated plus maze and open field tests [22, 24, 58, 59]. Accordingly, the effects of ELA observed here are likely due to differences in response to heroin rather than an anxiety response to a novel environment per se.

### Chronic opioid exposure might counteract some ELA-induced reward deficits

An intriguing trend in our data, though requiring replication in a larger sample, suggests that chronic opioid experience may counteract some of the effects of ELA on reward circuit responses to acute heroin, particularly in BLA. For example, ELA tended to alter overall BLA activation only in opioid-naïve rats. Likewise, ELA reduced activation of NAc-projecting BLA neurons in naïve rats, but this deficit was recovered following chronic opioid experience. This is consistent with this pathway’s reported reward-promoting function, and the fact that reduced activity in it results in anhedonia [90–93]. Such findings generally support the idea that anhedonia, such as that produced by ELA, may be “self-medicated” with opioid drugs [94, 95], and further work should be conducted to test this hypothesis.

Notably, the effects of ELA on heroin-induced BLA activity also align with evidence from humans and animals showing that ELA alters amygdala function and connectivity, particularly via projections to PFC [21, 96–101]. Considered alongside our current findings, the BLA-PFC pathway would be an intriguing target for future studies of ELA effects on an opioid reward.

### Experimental limitations and caveats

These studies delineate an ELA-induced behavioral phenotype consistent with anhedonia in male rats, as indicated by reduced heroin self-administration, and reduced remifentanil “hedonic setpoint.” These findings align with our prior work on the effects of ELA on intake and pursuit of other rewards [21, 22], whereas others showed that ELA via LBN may instead augment reward-seeking in male mice, including excessive alcohol intake [102]. Additionally, ELA imposed via other methods such as maternal separation tends to result in enhanced drug-seeking behaviors in males, as reviewed elsewhere [15, 103], thus highlighting the critical impact of adversity type and timing when it comes to influencing reward-related or emotional-like outcomes [104, 105].

Whereas we identified ELA-induced changes in neuronal activation in several reward and stress circuit nodes, we did not observe robust effects of ELA specifically on the predominantly glutamatergic inputs to NAc we studied. This may stem in part from the low number of subjects in some experiments. It is also possible that ELA preferentially affects non-NAc-projecting neurons in these regions (such as the BLA→PFC pathway known to be altered by ELA), or other glutamatergic or non-glutamatergic NAc inputs from stress or reward regions not included in the present study, such as dopaminergic inputs from ventral tegmental area. Additionally, only one subcutaneous dose of heroin was tested for these experiments; assessing effects of multiple doses administered either subcutaneously or intravenously (to more closely mimic the animal’s self-administered dosing) might reveal additional effects and would be instructive components of future studies.

It is also plausible that ELA differentially affects distinct cell types within a region. For example, our prior work showed that ELA alters CeA neuronal responses to rewards specifically in corticotropin-releasing hormone (CRH)-expressing neurons [21], and influenced gene expression selectively even in certain subpopulations of CRH-expressing neurons in the hypothalamus [106]. Alternatively, ELA may alter the abundance or survival of specific cell types [23, 58, 107]. These findings point to several important next steps for understanding the mechanisms by which disrupted reward-seeking occurs after ELA.

## Conclusions

Here we demonstrate that ELA alters the balance of stress and reward-related neuronal responses to heroin, and leads to behavioral disruptions consistent with anhedonia. Intriguingly, chronic exposure to heroin tends to mitigate some of the ELA-induced dampening of opioid-seeking behaviors and neuronal reward responses, leading to speculation that consumption of pro-hedonic opioids may counteract processes underlying the anhedonia-like phenotype provoked by ELA. Taken together with our previous work, our findings demonstrate that the effects of ELA on reward-seeking behaviors and neuronal responses are both sex- and reward-type specific. Further studies on how ELA alters the developmental trajectories of reward and stress circuits in both males and females will be critical for more completely understanding the relative risks for the myriad mental health consequences of ELA among men and women, how they may differ, and developing more effective and tailored interventions.

## Supplementary information

Supplemental Figure S1

Figure S1 legend
