# Supplementary material for: Enduring disruption of reward and stress circuit activities by early-life adversity in male rats
Source: Transl Psychiatry. 2022 Jun 16;12:251. doi: 10.1038/s41398-022-01988-w (PMC9200783; doi:10.1038/s41398-022-01988-w)
Supplement: Supplementary file 2 — Figure S1 legend [file 41398_2022_1988_MOESM2_ESM.docx]

**Figure S1.** *Correlation of Abcam (#GR3293718-1, 1:10,000) and Millipore (#ABE457, 1:5,000) rabbit anti-cFos antibodies.* (A) Average c-Fos density on neighboring tissue sections does not differ between antibodies. (B) cFos density per neighboring section is strongly correlated between both antibody types.
